# Life-Threatening Tracheal Obstruction from Mucosal Flap

**DOI:** 10.1093/icvts/ivaf220

**Published:** 2025-10-01

**Authors:** Alfonso Fiorelli, Gaetana Messina, Damiano Capaccio, Fausto Ferraro

**Affiliations:** Thoracic Surgery Unit, University of Campania “Luigi Vanvitelli”, Naples 80138, Italy; Thoracic Surgery Unit, University of Campania “Luigi Vanvitelli”, Naples 80138, Italy; Pneumology and Bronchoscopy Unit, Eboli Hospital, Salerno 84025, Italy; Anesthesiology Unit, University of Campania “Luigi Vanvitelli”, Naples 84025, Italy

**Keywords:** tracheal flap, rigid bronchoscopy, benign airway stenosis

## Abstract

Herein, we report an uncommon case of life-threatening airway obstruction that occurred in an 83-year-old man who underwent a difficult intubation and was extubated 48 hours later. After extubation, he experienced acute respiratory distress, stridor, and hypoxemia. Bronchoscopy evaluation showed a mobile tracheal flap causing severe obstruction and was followed by a rigid bronchoscopy with mechanical removal of the lesion and restored of airway patency. At 1-month follow-up, no recurrence was found.

## INTRODUCTION

Herein, we reported an uncommon case of life-threatening airway obstruction due to a mobile tracheal flap that occurred in an 83-year-old man who underwent a difficult intubation and was extubated 48 hours later. The lesion was successfully removed by rigid bronchoscopy, restoring airway patency.

## CASE PRESENTATION

**Figure 1.**
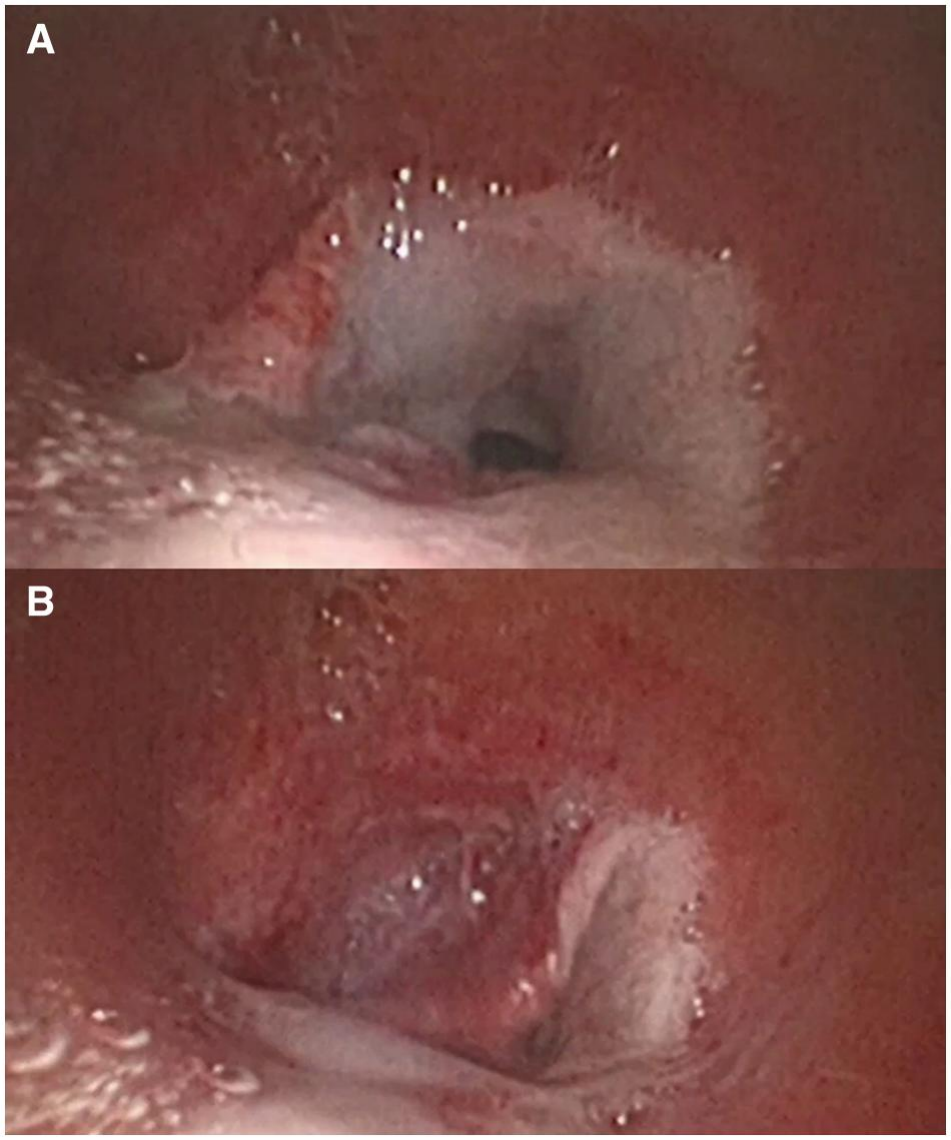
The Mobile Mucosal Flap (A) Caused Severe Tracheal Obstruction (B)

An 83-year-old male was referred to a local hospital for management of heart failure. Sudden hypoxemia needed emergent intubation and mechanical ventilation. A stylet was used for intubation due to the limited visualization of the vocal folds; then, resistance was encountered to the passage of the endotracheal tube through the airway. The patient’s clinical condition was stabilized; he was extubated 48 hours later, but experienced acute respiratory distress, stridor, and hypoxemia. This was initially thought to be secondary to mucous plug formation, but no improvement was obtained after repeated aspiration. The following bronchoscopy evaluation (Video 1) showed a mucosal flap (Figure 1A) that moved freely with the respiratory cycle and acted like a valve, causing severe tracheal obstruction (Figure 1B). Unsuccessful attempts were made to remove the flap during flexible bronchoscopy; the patient was re-intubated and transferred to our unit for the management.

**Figure 2.**
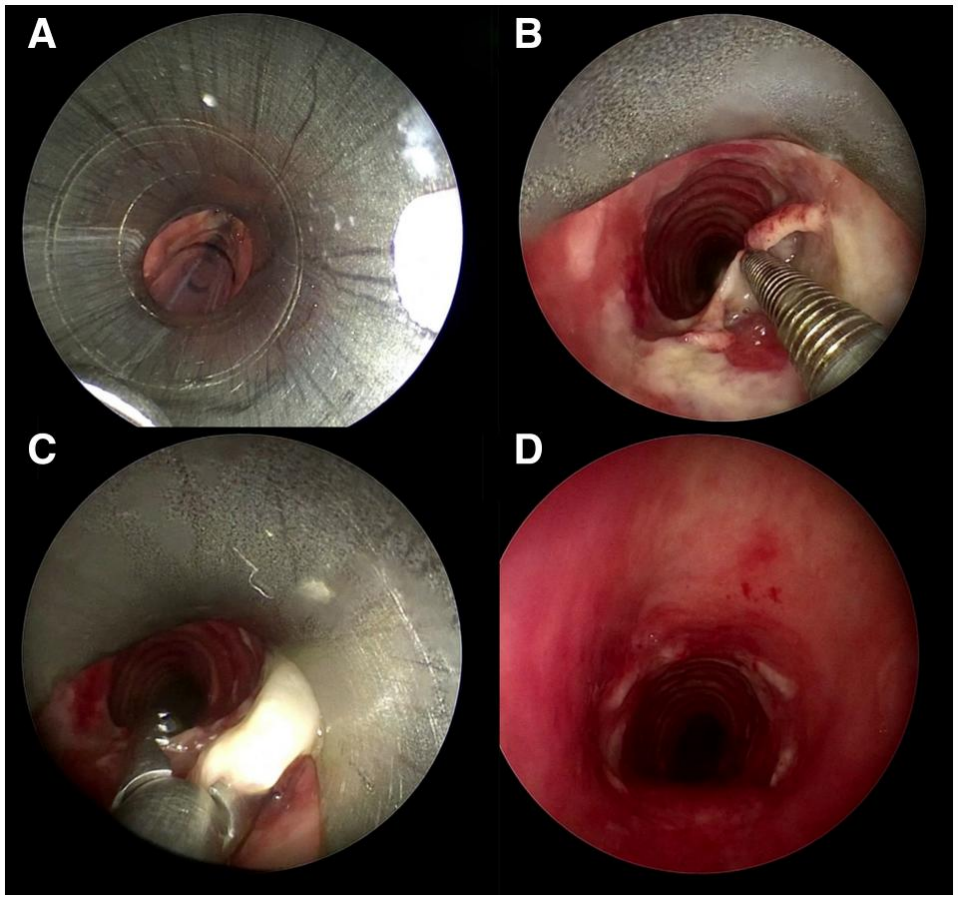
The Rigid Bronchoscopy Was Introduced Alongside the Indwelling Endotracheal Tube (A). The Flap Was Identified (B) and Removed by Alligator Forceps (C) Restoring the Airway Patency (D)

An emergent rigid bronchoscopy (RB) was performed (Video 2). An 8.5-mm outer diameter RB was introduced and advanced through the vocal cords into the larynx alongside the indwelling endotracheal tube (Figure 2A). The balloon cuff of the endotracheal tube was deflated, the tube removed as the RB carefully advanced into the trachea. Jet ventilation through the RB provided ventilation. The flap was identified (Figure 2B) and removed by alligator forceps restoring the airway patency (Figure 2C). The patient was extubated 24 hours later and transferred to the local hospital for ongoing cardiology care. At 1 month follow-up patient was well, and no recurrence of respiratory distress was found.

## DISCUSSION

The Obstructive Fibrinous Tracheal Pseudomembrane (OFTP) is a rare but potentially life-threatening cause of tracheal obstruction, typically occurring after prolonged endotracheal intubation, resulting from either mucosal injury during traumatic intubation. The ischaemia of the tracheal mucosa, inflammation, and necrosis, followed by the deposition of fibrin and inflammatory cells on the denuded mucosa evolves towards a firm pseudomembrane that causes fixed airway obstruction. Mechanical removal of the lesion by interventional bronchoscopic procedures allows restoring airway patency.^1–3^

In this case, the aetiology of mucosal flap was unclear. The short-term intubation, the thin characteristic of the lesion, and the valve-like mechanism of airway obstruction were in contrast with standard mechanism of OFTP. The resistance encountered during the initial intubation, as well as the organized, epithelialized appearance of the remnant flap observed during removal supports the hypothesis that tracheal flap results from a pre-existing tracheal web.^4^ Conversely, the absence of history of previous endotracheal intubation or tracheostomy and the lack of specific symptoms before intubation as acute stridor and sudden respiratory distress make also this hypothesis questionable. Thus, the iatrogenic damage caused by stylet remains the most likely explanation. Considering the difficult airway, the stylet was placed into the tracheal tube to curve it anteriorly and facilitate the passage through the vocal cords. Once it reached the trachea, the stylet teared the mucosa, resulting in a mobile flap that, following extubation acted as a valve, resulting in acute airway obstruction.

The flap was removed by RB to restore airway patency. The removal of a secure airway to place the RB could result in loss of airway. Thus, we used an RB of small size (8.5 diameter) that can pass alongside the tracheal tube through the vocal folds. Furthermore, the patient was not extubated until the RB was in the laryngeal inlet. Furthermore, the removal of tracheal flap could be associated with airway perforation, and a CT scan and/or bronchoscopic evaluation should be promptly performed if subcutaneous emphysema occurred after the procedure. In a similar case, Farr et al^5^ achieved a less invasive treatment by placing a 6.5-mm, uncuffed, nasotracheal tube beyond the lesion that remained in place for 1 week. When the tube was withdrawn, the mucosal flap appeared well adhered to the normal anatomical position with acceptable restore of airway patency. Significant risks of delayed obstruction and ICU-related morbidity were the main limitations of this strategy. In case of failure of mechanical removal of tracheal flap, tracheostomy is an available option to bypass stenosis and ensure ventilation.

In closure, this case underscores the importance of considering iatrogenic mucosal flap in the differential diagnosis of post-extubation respiratory stridor, especially in challenging airway cases. In such scenario, bronchoscopy evaluation followed by interventional bronchoscopic procedures allows an early diagnosis and a lifesaving treatment.

## Data Availability

The clinical data were available on request due to privacy.
